# Racial and Ethnic Disparities in U.S. Veteran Health Characteristics

**DOI:** 10.3390/ijerph18052411

**Published:** 2021-03-02

**Authors:** Rachel E. Ward, Xuan-Mai T. Nguyen, Yanping Li, Emily M. Lord, Vanessa Lecky, Rebecca J. Song, Juan P. Casas, Kelly Cho, John Michael Gaziano, Kelly M. Harrington, Stacey B. Whitbourne, on behalf of the VA Million Veteran Program

**Affiliations:** 1 Massachusetts Veterans Epidemiology Research and Information Center (MAVERIC), VA Boston Healthcare System, Boston, MA 02130, USA; Xuan-Mai.Nguyen@va.gov (X.-M.T.N.); Yanping.Li@va.gov (Y.L.); Emily.Lord@va.gov (E.M.L.); Vanessa.Lecky@va.gov (V.L.); Rebecca.Song@va.gov (R.J.S.); jpcasasromero@bwh.harvard.edu (J.P.C.); Kelly.Cho@va.gov (K.C.); Michael.Gaziano@va.gov (J.M.G.); Kelly.Harrington@va.gov (K.M.H.); Stacey.Whitbourne@va.gov (S.B.W.); 2 New England Geriatric Research, Education, and Clinical Center (GRECC), VA Boston Healthcare System, Boston, MA 02130, USA; 3 Carle Illinois College of Medicine, University of Illinois, Champaign, IL 61820, USA; 4 Department of Medicine, Harvard Medical School, Boston, MA 02115, USA; 5 Department of Epidemiology, Boston University School of Public Health, Boston, MA 02118, USA; 6 Department of Medicine, Division of Aging, Brigham and Women’s Hospital, Boston, MA 02115, USA; 7 Department of Psychiatry, Boston University School of Medicine, Boston, MA 02118, USA

**Keywords:** veterans, racial disparities, disease burden, healthcare utilization

## Abstract

Racial/ethnic health disparities persist among veterans despite comparable access and quality of care. We describe racial/ethnic differences in self-reported health characteristics among 437,413 men and women (mean age (SD) = 64.5 (12.6), 91% men, 79% White) within the Million Veteran Program. The Cochran–Mantel–Haenszel test and linear mixed models were used to compare age-standardized frequencies and means across race/ethnicity groups, stratified by gender. Black, Hispanic, and Other race men and women reported worse self-rated health, greater VA healthcare utilization, and more combat exposure than Whites. Compared to White men, Black and Other men reported more circulatory, musculoskeletal, mental health, and infectious disease conditions while Hispanic men reported fewer circulatory and more mental health, infectious disease, kidney, and neurological conditions. Compared to White women, Black women reported more circulatory and infectious disease conditions and Other women reported more infectious disease conditions. Smoking rates were higher among Black men, but lower for other minority groups compared to Whites. Minority groups were less likely to drink alcohol and had lower physical fitness than Whites. By identifying differences in burden of various health conditions and risk factors across different racial/ethnic groups, our findings can inform future studies and ultimately interventions addressing disparities.

## 1. Introduction

A major goal of the Veterans Health Administration (VA) is to become a leader in addressing health disparities and achieving equity in healthcare and health outcomes among vulnerable populations. The VA healthcare system is the largest integrated healthcare system in the United States, with over 9 million veteran enrolled. More than 20% of VA healthcare users are members of racial and ethnic minority groups with 16% identifying as Black or African-American and 5% identifying as Hispanic [1]. Access to care within the VA healthcare system is dependent on service-connected disability and other eligibility criteria that are perhaps less impacted by socioeconomic factors than access to healthcare within the private sector. However, racial and ethnic health disparities are still present within the VA.

Research within the VA has shown that racial and ethnic disparities in clinical outcomes and rates of chronic disease among veterans persist even when access and quality of care are comparable [2,3]. VA healthcare system data from 2000–2009 showed that despite improvements in equality of process of care measures (i.e., receiving recommended care), Black veterans experienced worse control of cholesterol, blood pressure, and glucose than White veterans within the same facilities [2]. Similarly, racial and ethnic disparities in chronic conditions have been observed. Longitudinal evidence has shown that minority veterans with diabetes are more likely to experience early nephropathy, but less likely to experience cardiovascular disease than their White counterparts [3]. These findings suggest that access and equality of care do not completely account for observed racial and ethnic health disparities. Therefore, in order to appropriately tackle existing disparities, a better understanding of the burden of various health conditions and underlying risk factors among veterans of different racial and ethnic groups is needed.

While prior work has identified racial and ethnic disparities in some clinical outcomes and health conditions among veterans, several limitations exist. Despite evidence from the VA Million Veteran Program (MVP) and other studies that veteran women have higher rates of certain mental and physical health conditions [4,5], few have investigated racial and ethnic disparities among veterans by gender [6], particularly for physical health conditions. In addition, studies on differences in potential risk factors for health conditions among veterans of different races and ethnicities have been limited. While there is some evidence that differences in smoking rates may account for some of the racial health disparity, particularly among Black veteran men [7], other lifestyle risk factors such as physical fitness and exercise have been largely unexplored. In addition, differences in combat exposure have been found to explain some of the relationship between race and health disparities among veteran men [7]; however, it is unknown whether combat exposure plays a similar role among veteran women. 

The primary aim of this research was to describe racial and ethnic differences in health-related characteristics among men and women in MVP. We will explore racial and ethnic differences in the following health-related characteristics: (1) self-rated health status; (2) number of lifetime health conditions; (3) healthcare utilization; (4) lifestyle risk factors; and (5) military exposures, including combat.

## 2. Materials and Methods

The Million Veteran Program (MVP) is an ongoing observational cohort of US veterans designed to investigate how health is affected by genetics, behavior, and environmental factors. A detailed description of the cohort design has been published previously [8]. MVP includes a collection of self-reported survey information, biospecimen data, and access to health record data. All participants were regular users of the VA Healthcare System. Out of 800,207 participants enrolled between January 2011 and November 2019, we included data from 437,413 participants without missing race and ethnicity data who completed the MVP Baseline and Lifestyle Surveys. The study was conducted according to the guidelines of the Declaration of Helsinki and approved by the VA Central Institutional Review Board (protocol code: 10-02; date of approval: 2010). Informed consent was obtained from all participants.

The MVP Baseline and Lifestyle Survey instruments have been previously published in their entirety [8]. Briefly, the surveys contain items assessing demographics, health status, medical history, family medical history, lifestyle behaviors, and military experiences. Data for this analysis were taken from both surveys with a few exceptions. If date of birth and/or gender were missing, they were obtained from the electronic health record. Some response categories were combined due to small numbers among a few groups.

Race was assessed with the question “What is your race?”, and responses included the following: White, Black/African-American, American Indian/Alaska Native, Chinese, Japanese, Asian Indian, Other Asian, Filipino, Pacific Islander, and Other. Ethnicity was assessed with the question “Are you Spanish, Hispanic, or Latino?” Responses included “No, not Spanish, Hispanic, Latino,” “Yes, Mexican, Mexican American, Chicano,” “Yes, Puerto Rican,” “Yes, Cuban,” and “Yes, other Spanish, Hispanic, Latino.” Race and ethnicity were combined into the following categories: White (Non-Hispanic), Black (Non-Hispanic), Hispanic, and Other.

Additional demographic and lifestyle risk factors included education level (≥some college, yes/no), marital status (married or cohabitating with partner, yes/no) and annual household income (<$30,000, $30,000–$59,999, $60,000–$99,999, and ≥$100,000). Current physical fitness (very good to fairly good, satisfactory, and fairly poor to very poor) and exercise frequency (≤1–3 times/month, once/week, 2–4 times/week, and ≥5 times/week) were also assessed. Smoking status and alcohol use were categorized as “never”, “current”, and “former” as described previously [9].

Service era was reported and included the categories September 2001 or later, August 1990 to August 2001 (includes Gulf War), May 1975 to July 1990, August 1964 to April 1975 (Vietnam era), February 1955 to July 1964, July 1950 to January 1955 (Korean War), January 1947 to June 1950, December 1941 to December 1946 (WWII), and November 1941 or earlier. Categories for branch of services included Army, Navy, Air Force, Marine Corps, and Other. Military exposures including combat exposure, traumatic brain injury (TBI), and chemical or biological warfare agent exposures were measured using the Combat Experiences Scale from the Deployment Risk and Resilience Inventory (DRRI) [10], The Brief Traumatic Brain Injury Screen (BTBIS) [11], and the adapted Structured Neurotoxicant Assessment Checklist (SNAC) [12], respectively.

Self-rated health status was assessed by asking “In general, would you say your health is: excellent, very good, good, fair, or poor.” Categories were collapsed into good to excellent and fair to poor. Pain intensity within the past week was assessed using standard cut-points (0 = no pain, 1–3 = mild pain, 4–6 = moderate pain, and 7–10 = severe pain) [13]. Participants were also asked if they had ever been diagnosed with 75 individual conditions within the following categories: circulatory system, mental health, skeletal/muscular, hearing/vision, infectious disease, cancer, kidney disease, digestive system, nervous system, and other. Individual conditions listed by category have been previously published [8]. The number of lifetime health conditions overall and in each category were summed. Healthcare utilization was assessed as proportion of healthcare received at a VA facility in the past year (none, less than half of care, more than half of care, and all care), number of inpatient stays in the past year (none, 1–3, and ≥4), and current number of prescription medications received at both VA and Non-VA pharmacies, separately (1–3, 4–6, and ≥7).

Continuous variables were reported as means and standard deviations and categorical variables as percentages. Direct standardization was used to standardize demographics and health-related characteristics to the age distribution of Non-Hispanic White participants [4,14,15]. Age groups were stratified into 10-year increments (e.g., 30–39 years) except for the youngest (18–29 years) and the oldest (≥80 years) age groups. Standardization was performed separately for men and women. Age-adjusted characteristics for Non-Hispanic Black men, Hispanic men, and men in the Other race/ethnicity category were compared with those for Non-Hispanic White men, separately, using the Cochran–Mantel–Haenszel test for frequencies and a linear mixed model for means. Age-adjusted means and frequencies among women were compared similarly. The Bonferroni correction was used to account for multiple comparisons. Applying a desired alpha level of 0.05 with a total of 126 comparisons, a corrected alpha level of 0.0004 was used. Analyses were performed using SAS software version 9.4 (SAS Institute Inc., Cary, NC, USA).

## 3. Results

### 3.1. Demographics

Among 437,413 participants, the mean age (SD) was 64.5 (12.6) and 91% were men. Among men, 80% were White, 11% were Black, 7% were Hispanic, and 2% were Other race/ethnicity. Among women, 68% were White, 20% were Black, 9% were Hispanic, and 3% were Other race/ethnicity.

Age-adjusted demographics, lifestyle characteristics, and military exposures stratified by race and ethnicity are presented for men and women in Table 1 and Table 2, respectively. Compared to White men and women, Black, Hispanic, and Other men and women were younger (66.8 vs. 60.7, 60.9, and 60.3 years, respectively for men; 55.0 vs. 51.8, 48.1, and 50.1 years, respectively for women). Compared to White men, Black and Hispanic men were less likely to be married or cohabitating with a partner, less likely to have had some college, and tended to be in lower household income categories. While marital status and household income did not differ between White and Other men, Other men were more likely to have some college. Compared to White women, Black and Hispanic women were less likely to be married or cohabitating with a partner and Black women tended to be in lower household income categories. Rates of having some college did not differ among racial/ethnic groups in women. 

### 3.2. Lifestyle Characteristics

Compared to White men, Black men were more likely to be current smokers and Hispanic and Other men were less likely to be former smokers. Black, Hispanic, and Other men were less likely to currently drink alcohol and more likely to report fairly poor to very poor physical fitness than White men. While Black men reported less frequent exercise than White men, Hispanic men reported slightly more. Compared to White women, Black and Hispanic women were less likely to be current and former smokers and Other women were less likely to be former smokers. Black, Hispanic, and Other women were more likely to have never used alcohol than White women. Among women, physical fitness did not differ by race/ethnicity group; however, Black women reported less frequent exercise than White women.

### 3.3. Military Service and Combat Exposures

Except for Black men, minority groups were more likely to serve during the Gulf War and after (August 1990 to present), compared to White veterans. Black men had the highest rates of serving in the Army and the Air Force (59.0% and 18.9% respectively), while Other men had the highest rate of serving in the Navy (27.2%), and Hispanic men had the highest rate of serving in the Marine Corps (14.4%). Rates of service among the different military branches were similar for women, except White women had the highest rate of serving in the Air Force (25.8%) and Other women had the highest rate of serving in the Marine Corps (10.9%).

Compared to White men, Black, Hispanic and Other men had higher scores on the DRRI Combat Experiences Scale (25.0 (11.3) vs. 26.1 (13.2), 27.5 (13.3), and 26.1 (13.0)) and were more likely to have a positive screen for TBI on the BTBIS (25.3% vs. 29.0%, 36.4%, 37.0%, all *p* < 0.0001). Black and Hispanic men were less likely to report exposure to chemical or biological warfare agents than White men (76.2% and 78.6% vs. 81.6%, both *p* < 0.0001). Among women, scores on the Combat Experiences Scale did not significantly differ by racial/ethnic group. Compared to White women, Black and Hispanic women were more likely to screen positive for TBI on the BTBIS (15.1% vs. 19.7% and 24.2%, *p =* 0.0002 and *p* < 0.0001, respectively). Other women also had higher rates of positive TBI screen on the BTBIS than White women; however, differences did not reach statistical significance after correction for multiple comparisons (26.4% vs. 15.1%, *p =* 0.0019). Compared to White women, Other women were more likely to report exposure to chemical or biological warfare agents (74.0% vs. 65.2%, *p* < 0.0001).

### 3.4. Health-Related Characteristics

Health-related characteristics among different racial/ethnic groups are presented in Table 3 and Table 4 for men and women, respectively. Compared to White men, Black, Hispanic, and Other men were more likely to report fair to poor current health status (36.9% vs. 48.8%, 53.3%, and 53.6%) and severe pain intensity in the past week (18.7% vs. 34.6%, 30.5%, and 30.4%; all *p* < 0.0001). Black men reported fewer total lifetime health conditions relative to White men, while Hispanic and Other men reported more (mean (SD): 7.42 (5.11), 7.82 (5.35), and 8.33 (5.62) vs. 7.52 (4.60); all *p* < 0.0001). Compared to White men, Black, Hispanic, and Other men had more mental health, infectious disease, and kidney conditions, and fewer gastrointestinal conditions and cancers (all *p* < 0.0001). Compared to White men, Black and Other men had more circulatory and skeletomuscular conditions (all *p* < 0.0001). Compared to White men, Hispanic men had fewer circulatory conditions (*p =* 0.0003). Black men reported fewer hearing and vision conditions than White men, while Other men reported more (both *p* < 0.0001). Compared to White men, Black men had fewer neurological conditions, while Hispanic and Other men had more (both *p* < 0.0001). Black and Hispanic men were more likely than White men to receive all of their healthcare from the VA, while Other men were less likely (43.4%, 40.4%, and 35.0% vs. 39.6%; all *p* < 0.0001). Compared to White men, Black, Hispanic and Other men were more likely to report inpatient stays within the past year and more prescription medications received both from and outside of the VA (all *p* < 0.0001).

Similar to men, Black, Hispanic, and Other women were more likely to report fair to poor current health status (40.5%, 39.6%, and 38.5% vs. 32.7%) and severe pain over the past week (38.8%, 33.9%, and 36.0% vs. 25.7%; all *p* < 0.0001) than White women. Compared to White women, Black women reported fewer total lifetime health conditions, (6.53 (4.42) vs. 7.17 (4.70)) skeletomuscular, mental health, hearing and vision, gastrointestinal, and neurological conditions as well as cancers (all *p* < 0.0001). Black women reported more circulatory conditions and infectious diseases than White women (both *p* < 0.0001). Hispanic and Other women reported fewer cancers than White women (both *p* < 0.0001). Compared to White women, Hispanic women also reported fewer gastrointestinal conditions (*p* < 0.0001), while Other women reported more infectious diseases (*p =* 0.0002). Hispanic women were less likely than White women to receive all of their healthcare from the VA (42.8% vs. 45.5%; *p* < 0.0001). Compared to White women, Black and Hispanic women were more likely to report VA inpatient stays within the past year (*p* < 0.0001 and *p =* 0.0002). Black women reported more prescription medications received from the VA and outside of the VA than White women (both *p* < 0.0001).

## 4. Discussion

While access to care within the VA healthcare system is dependent on service-connected disability and other eligibility criteria that are likely less impacted by socioeconomic factors than access to healthcare within the private sector, we observed a number of different racial and ethnic disparities, many of which have previously been observed among non-veteran populations [16,17]. In addition, we observed disparities in potential health-related risk factors that may be specific to or more prevalent in the veteran population, such as combat exposure and exposure to biological and chemical warfare agents. Gaining a better understanding of differences in burden of various health conditions and risk factors across different racial/ethnic groups is an important step toward achieving health equity within the veteran population and in general.

Among US veterans in the Million veteran Program, Black, Hispanic, and Other race/ethnicity men and women reported worse self-rated health than White men and women. Racial/ethnic disparities in burden of disease varied greatly by disease category. Overall, those from minority racial/ethnic groups reported lower markers of socioeconomic status (i.e., marital status, education, and income), more combat exposure, more positive TBI screens, and greater VA healthcare utilization. 

Our findings are consistent with and expand findings from previous literature and reports. Among 6998 male veterans ages 30 to 84 from the 2010 National Survey of veterans (NSV), Sheehan et al. found that Black, Hispanic and Other/multiple race veteran men were more likely to report fair to poor self-rated health than White veterans (NSV unadjusted rates: 44.1%, 30.6%, and 33.1%, vs. 25.4%; *p* < 0.05) [7]. Our study confirms these findings in a large sample of men and extends them to veteran women.

The National Veterans Health Equity Report, which used administrative data to examine differences in the prevalence of health conditions among different racial/ethnic groups, found that similar to our findings, Black and Hispanic veterans were more likely to be diagnosed with mental health, musculoskeletal, and infectious disease conditions and less likely to be diagnosed with cancer than White veterans [1]. Hispanic veterans had a lower rate of cardiovascular conditions and Black veterans had lower rates of sense organ conditions than White veterans. Contrary to our findings, they found that Black and Hispanic veterans had similar rates of gastrointestinal and neurologic conditions and that Black veterans had a similar rate of cardiovascular conditions to White veterans. It is important to note that data presented in the National Veterans Health Equity Report was unadjusted. Therefore, it is not possible to distinguish whether differences in disease rates were due to differences in age distributions among the different racial/ethnic categories. Since minority veterans tend to be younger, not adjusting for age could explain why they did not observe higher rates of cardiovascular, gastrointestinal, and neurological conditions in minority veterans compared to White veterans. In addition, this report looked at prevalence of any condition within disease categories whereas our study looked at the number of conditions within each disease category. This could also account for some of the differences observed.

It has been suggested that differences in smoking rates may account for some of the health disparities among minority veterans. The NSV found, consistent with our findings, that Black veteran men had a higher rate of current smoking than White veteran men (NSV unadjusted rates: 35.7% vs. 17.9%) [7]. While the NSV found that this difference in smoking rate did not account for disparities in self-reported health, they did not look at specific categories of conditions. It is possible that the higher smoking rates we observed among Black veteran men compared to White veteran men may have contributed to the greater number of circulatory conditions we observed among Black veteran men. Both the NSV and our study found that Hispanic veteran men had higher rates of never smoking than White veteran men (NSV unadjusted rates: 46.1% vs. 33.1%) and that Other veteran men were less likely to be former smokers but more likely to be current smokers than White veteran men (NSV unadjusted rates: 31.9% vs. 49.0% and 28.7% vs. 17.9%, respectively). Interestingly, within the NSV, they found that adjusting for smoking actually widened the disparity for self-reported health between Hispanic veteran men and White veteran men (NSV OR and p-value for model with smoking vs. without: 1.47, *p* < 0.01 vs. 1.37, *p* < 0.05). We additionally found that minority veteran women were less likely to be former and current smokers than White veteran women. While there is limited research on smoking rates among veteran women by racial/ethnic group, our findings are in contrast with two previous studies that have found no differences in current smoking status among White and Black veteran women [18,19]. This difference could be due to the smaller sample sizes or lack of adjustment for age in previous studies, since current smoking status rates tend to decrease with age [20].

Literature on racial/ethnic differences among veterans in other behavioral/lifestyle factors such as alcohol consumption, exercise frequency, and physical fitness is lacking. We found that minority veteran men and women were more likely to report never drinking alcohol than White veteran men and women. Minority veteran men were more likely to report fairly poor to very poor physical fitness. We found no differences in self-reported physical fitness among women of different racial/ethnic groups. While Black veteran men and women tended to report less frequent exercise than White veteran men and women, Hispanic veteran men tended to report more exercise. veterans from each minority group were more likely to report severe pain in the past week than White veterans. This finding is consistent with two studies that found that Black veterans are more likely to experience more severe osteoarthritis symptoms [21] and chronic pain [22] compared to White veterans. Moreover, racial disparities in both screening and treatment of pain have been identified within the VA, with Black veterans being less likely to be screened for pain [23] and less likely to receive adequate pain treatment [24,25].

Another potential source of health disparities among minority veterans may be differences in combat exposure. Among veteran men, combat exposure has been linked to a variety of health conditions, including PTSD, arthritis, pain, headaches, asthma, lung disease, heart disease, and stroke [26,27,28,29,30]. Higher rates of disease have been associated with wartime stressors that occurred decades prior [29]. Higher rates of PTSD among Black and Hispanic veterans have been reported [31] and may be explained by higher rates of combat exposure [6,31]. Importantly, the NSV found that differences in military experience factors accounted for a portion of the racial/ethnic disparities in self-reported health and activity limitations beyond conventional socioeconomic and behavioral factors. It is important to note that this study included men only [7]. Similar to findings from the NSV, we observed that minority veteran men were more likely than White veteran men to report combat exposure. Data from the NSV also showed that some of the differences in military exposures may be due to minority veterans being more likely to serve in the Army than White veterans, which is consistent with the differences we found among men. While minority women were also more likely to serve in the Army than White women, we found no differences among women for combat exposure reported from the DRRI. This may in part be explained by the fact that combat roles were opened to women in 2015 [32]. Additional work will explore this finding in more detail among recently returned veterans. 

We did find that minority veteran women and men were more likely to experience TBI than White veteran men and women. Much of the previous literature has focused on racial/ethnic disparities in severity or outcomes among veterans with TBI rather than racial/ethnic differences in the occurrence of TBI [33,34,35]. However, a study of 170,681 Operation Enduring Freedom and/or Operation Iraqi Freedom (OEF/OIF) veterans who received care at VA medical facilities from 2007 to 2008 found that Black veterans had lower rates of positive initial screens for TBI than White veterans [36]. They found no differences in rates of positive TBI screens for Hispanic or Other race veterans compared to White veterans when adjusted for sociodemographic and military-service related factors.

Contrary to our findings, data from the NSV found that minority veterans were more likely to report exposure to environmental hazards [7]. Within our study, Black and Hispanic veteran men were less likely to be exposed to chemical or biological warfare agents than White veteran men, while Other veteran men and women were more likely to be exposed than White veteran men and women. We found no differences between Black and Hispanic veteran women and White veteran women in chemical or biological warfare agent exposure. More work is needed in this area.

Our research has several strengths. We had detailed self-reported data on a large number of veterans, which allowed us to investigate racial disparities separately for men and women. To our knowledge, this is the largest study that includes data on lifestyle risk factors in U.S. veterans. Bonferroni correction was used as a conservative approach to reduce the likelihood of Type I error, given the large sample size. Despite using this approach, we observed significant differences between racial/ethnic groups in both men and women. Limitations of our study include that we did not look at rates of specific diseases or severity of disease. We also had fewer numbers of veteran women than men. It is possible that this may have contributed to the fewer significant differences we observed among women. We also did not evaluate length of service or military rank.

## 5. Conclusions

Our study identified several racial/ethnic disparities in disease burden and other health-related factors as well as potential risk factors. These findings can provide the VA with a better understanding of differences in disease burden and risk factors among racial and ethnic groups to address health disparities and achieve equity in healthcare and health outcomes. Future work will further investigate the complex relationships between these risk factors and health disparities to inform interventions and policy changes that will better serve minority veteran men and women.

## Figures and Tables

**Table 1:** Demographics, Lifestyle Characteristics, and Military Exposures of Men from the Million Veteran Program by Race and Ethnicity.

Characteristic	Unadjusted	Age-Standardized					
	White	Black	*p*-Value	Hispanic	*p*-Value	Other	*p*-Value
	(*n* = 317,782)	(*n* = 42,951)		(*n* = 29,427)		(*n* = 8782)	
**Age, years, mean (SD)**	66.8 (11.7)	60.7 (10.6)		60.9 (13.6)		60.3 (13.7)	
Age category (%)							
18–45	5.30	7.50		13.93		15.29	
>45–64	29.69	56.41		41.49		41.58	
≥65	65.02	36.09		44.58		43.14	
Married or cohabitating with partner (%)	66.21	51.96	<0.0001	61.99	<0.0001	65.00	0.0021
Education level, ≥some college (%)	75.22	70.34	<0.0001	69.39	<0.0001	81.92	<0.0001
Annual household income (%)			<0.0001		<0.0001		0.56962
<$30,000	33.19	44.08		40.25		33.06	
$30,000–$59,999	35.66	32.58		35.83		34.82	
$60,000–$99,999	19.97	16.79		16.80		20.10	
≥$100,000	11.17	6.55		7.11		12.02	
Smoking status (%)			<0.0001		<0.0001		<0.0001
Never	26.3	27.0		32.6		28.1	
Former	67.7	66.6		61.5		65.8	
Current	6.0	6.4		5.9		6.1	
Alcohol use (%)			<0.0001		<0.0001		<0.0001
Never	7.3	8.8		8.0		11.6	
Former	37.1	47.8		43.5		44.1	
Current	55.6	43.4		48.5		44.3	
Current physical fitness status (%)			<0.0001		<0.0001		<0.0001
Very good to fairly good	39.1	30.6		32.3		31.8	
Satisfactory	34.0	37.0		36.1		36.4	
Fairly poor to very poor	26.9	32.3		31.6		31.7	
Current exercise frequency (%)			<0.0001		<0.0001		0.0008
≤1–3 times/month	44.3	47.9		42.9		42.8	
Once/week	13.3	13.3		14.2		14.2	
2–4 times/week	28.4	27.1		28.3		28.4	
≥5 times/week	14.0	11.7		14.6		14.6	
Service era (%)			<0.0001		<0.0001		<0.0001
September 2001 or later	9.2	8.1		9.9		11.4	
August 1990 to August 2001 (includes Gulf War)	15.0	15.2		15.4		19.9	
May 1975 to July 1990	24.5	26.2		22.6		31.2	
August 1964 to April 1975 (Vietnam era)	58.5	58.1		56.2		58.5	
February 1955 to July 1964	17.6	15.7		14.8		14.2	
July 1950 to January 1955 (Korean War)	9.9	10.0		10.5		9.3	
January 1947 to June 1950	1.5	1.4		1.4		1.3	
December 1941 to December 1946 (WWII)	4.1	3.0		3.0		2.9	
November 1941 or earlier	0.2	0.2		0.2		0.1	
Branch of service (%)			<0.0001		<0.0001		<0.0001
Army	47.3	59.0		56.8		48.3	
Navy	24.1	13.0		16.2		27.2	
Air Force	18.6	18.9		14.8		15.7	
Marine Corps	11.5	11.0		14.4		10.9	
Other	7.7	5.3		8.3		7.7	
Military Exposures							
Combat experiences scale ^1^ (15–75) mean (SD)	25.0 (11.3)	26.1 (13.2)	<0.0001	27.5 (13.3)	<0.0001	26.1 (13.0)	<0.0001
Brief Traumatic Brain Injury Screen (%)	25.3	29.0	<0.0001	36.4	<0.0001	37.0	<0.0001
Chemical or biological warfare agent exposure ^2^ (%)	81.6	76.2	<0.0001	78.6	<0.0001	83.2	0.0681

^1^ Combat Experiences Scale from the Deployment Risk and Resilience Inventory. ^2^ Using an adapted Structured Neurotoxicant Assessment Checklist.

**Table 2:** Demographics, Lifestyle Characteristics, and Military Exposures of Women from the Million Veteran Program by Race and Ethnicity.

Characteristic	Unadjusted	Age-Standardized					
	White	Black	*p*-Value	Hispanic	*p*-Value	Other	*p*-Value
	(*n* = 26,139)	(*n* = 7632)		(*n* = 3482)		(*n* = 1218)	
**Age, years, mean (SD)**	55.0 (13.4)	51.8 (10.7)		48.1 (13.6)		50.1 (12.7)	
Age category (%)							
18–45	22.79	25.84		43.44		35.63	
>45–64	55.45	65.23		45.76		51.89	
≥65	21.76	8.92		10.80		12.48	
Married or cohabitating with partner (%)	42.97	28.11	<0.0001	36.88	<0.0001	43.17	0.79153
Education level, ≥some college (%)	90.76	92.41	0.00618	90.80	0.54979	93.98	0.00169
Annual household income (%)			<0.0001		0.03078		0.78796
<$30,000	34.21	37.03		34.03		34.38	
$30,000–$59,999	33.33	35.31		35.38		32.24	
$60,000–$99,999	19.44	18.67		19.57		19.23	
≥$100,000	13.03	8.99		11.02		14.15	
Smoking status (%)			<0.0001		<0.0001		0.0002
Never	39.7	49.5		49.4		44.4	
Former	54.1	44.9		44.9		49.4	
Current	6.2	5.6		5.6		6.2	
Alcohol use (%)			<0.0001		<0.0001		<0.0001
Never	9.5	15.4		15.6		20.4	
Former	33.8	35.2		29.3		34.4	
Current	56.7	49.4		55.1		45.2	
Current physical fitness status (%)			0.0017		0.0006		0.8645
Very good to fairly good	34.5	30.8		31.7		33.8	
Satisfactory	33.7	37.7		35.6		33.5	
Fairly poor to very poor	31.8	31.4		32.7		32.6	
Current exercise frequency (%)			<0.0001		0.0195		0.0024
≤1–3 times/month	47.7	49.9		43.8		43.1	
Once/week	13.3	13.0		14.8		12.6	
2–4 times/week	27.9	27.7		30.5		31.3	
≥5 times/week	11.1	9.4		10.9		12.9	
Service era (%)			<0.0001		<0.0001		<0.0001
September 2001 or later	28.6	31.3		34.1		36.2	
August 1990 to August 2001 (includes Gulf War)	40.5	47.2		41.2		42.6	
May 1975 to July 1990	49.4	49.5		44.5		47.2	
August 1964 to April 1975 (Vietnam era)	25.1	18.7		21.0		20.7	
February 1955 to July 1964	4.8	3.8		5.0		4.0	
July 1950 to January 1955 (Korean War)	2.2	2.5		2.1		1.4	
January 1947 to June 1950	0.2	0.2		0.0		0.0	
December 1941 to December 1946 (WWII)	1.3	0.6		0.8		0.6	
November 1941 or earlier	0.04	0.04		0.1		0.0	
Branch of service (%)			<0.0001		<0.0001		<0.0001
Army	43.7	60.1		51.3		48.3	
Navy	24.1	15.3		20.0		27.2	
Air Force	25.8	20.1		21.0		15.7	
Marine Corps	6.5	4.2		7.5		10.9	
Other	9.0	7.8		8.5		7.7	
Military Exposures							
Combat experiences scale ^1^, (15–75) mean (SD)	19.4 (7.43)	20.0 (8.74)	0.0021	21.2 (7.54)	0.0005	20.3 (9.49)	0.0563
Brief Traumatic Brain Injury Screen (%)	15.1	19.7	0.0002	24.2	<0.0001	26.4	0.0019
Chemical or biological warfare agent exposure ^2^ (%)	65.2	62.5	0.0033	66.3	0.2722	74.0	<0.0001

^1^ Combat Experiences Scale from the Deployment Risk and Resilience Inventory. ^2^ Using an adapted Structured Neurotoxicant Assessment Checklist.

**Table 3:** Health-related Characteristics of Men from the Million Veteran Program by Race and Ethnicity.

Characteristic	Unadjusted	Age-Standardized					
	White	Black	*p*-Value	Hispanic	*p*-Value	Other	*p*-Value
	(*n* = 317,782)	(*n* = 42,951)		(*n* = 29,427)		(*n* = 8782)	
Current health status (%)			<0.0001		<0.0001		<0.0001
Good to excellent	63.1	48.8		53.3		53.6	
Fair to poor	36.9	51.2		46.7		46.4	
Lifetime health conditions (No.), mean (SD)							
Total health conditions (0–75)	7.52 (4.60)	7.42 (5.11)	<0.0001	7.82 (5.35)	<0.0001	8.33 (5.62)	<0.0001
Circulatory conditions (0–10)	1.82 (1.49)	1.88 (1.44)	<0.0001	1.79 (1.49)	0.0003	1.91 (1.55)	<0.0001
Skeletomuscular conditions (0–6)	0.79 (0.90)	0.85 (0.98)	<0.0001	0.80 (0.97)	0.9270	0.95 (1.06)	<0.0001
Mental health conditions (0–10)	0.70 (1.21)	0.90 (1.43)	<0.0001	1.01 (1.52)	<0.0001	0.93 (1.49)	<0.0001
Hearing and vision conditions (0–6)	1.16 (1.08)	0.88 (1.07)	<0.0001	1.14 (1.14)	0.0074	1.24 (1.17)	<0.0001
Infectious diseases (0–4)	0.08 (0.29)	0.16 (0.42)	<0.0001	0.12 (0.37)	<0.0001	0.13 (0.37)	<0.0001
Kidney conditions (0–3)	0.07 (0.28)	0.12 (0.38)	<0.0001	0.10 (0.34)	<0.0001	0.11 (0.35)	<0.0001
Gastrointestinal conditions (0–9)	0.75 (0.89)	0.64 (0.90)	<0.0001	0.72 (0.95)	<0.0001	0.73 (0.97)	<0.0001
Cancers (0–6)	0.39 (0.62)	0.26 (0.51)	<0.0001	0.22 (0.50)	<0.0001	0.25 (0.52)	<0.0001
Neurological conditions (0–12)	0.56 (1.01)	0.52 (0.99)	<0.0001	0.63 (1.13)	<0.0001	0.71 (1.19)	<0.0001
Other conditions (0–9)	1.19 (1.15)	1.22 (1.18)	0.0231	1.30 (1.21)	<0.0001	1.37 (1.25)	<0.0001
Pain intensity, past week (%)			<0.0001		<0.0001		<0.0001
No pain (0)	12.6	10.6		11.0		9.8	
Mild pain (1–3)	40.8	24.5		27.6		29.9	
Moderate pain (4–6)	27.9	30.3		31.0		29.8	
Severe pain (7–10)	18.7	34.6		30.5		30.4	
VA health care use, past year (%)			<0.0001		<0.0001		<0.0001
None	9.3	7.7		9.2		10.0	
Less than half of care	27.4	22.8		28.4		31.0	
More than half of care	23.8	26.0		22.1		24.0	
All care	39.6	43.4		40.4		35.0	
VA inpatient hospital stays, past year (%)			<0.0001		<0.0001		<0.0001
None	81.7	73.4		75.1		77.6	
1–3	15.3	21.1		19.6		17.7	
≥4	3.0	5.6		5.3		4.7	
Current prescription medications from VA (%)			<0.0001		<0.0001		<0.0001
None	16.6	9.9		13.2		15.6	
1–3	29.3	22.4		26.8		27.4	
4–6	26.8	29.0		27.7		26.3	
≥7	27.2	38.8		32.4		30.7	
Current prescription medications outside VA (%)			<0.0001		<0.0001		<0.0001
None	40.9	39.4		37.2		34.2	
1–3	34.4	32.6		33.6		33.1	
4–6	16.4	17.0		18.3		19.3	
≥7	8.3	11.1		10.9		13.3	

No. = number.

**Table 4:** Health-related Characteristics of Women from the Million Veteran Program by Race and Ethnicity.

Characteristic	Unadjusted	Age-Standardized					
	White	Black	*p*-Value	Hispanic	*p*-Value	Other	*p*-Value
	(*n* = 26,139)	*(n* = 7632)		(*n* = 3482)		(*n* = 1218)	
Current health status (%)			<0.0001		<0.0001		<0.0001
Good to excellent	67.3	59.5		60.4		61.5	
Fair to poor	32.7	40.5		39.6		38.5	
Lifetime health conditions (No.), mean (SD)							
Total health conditions (0–75)	7.17 (4.70)	6.53 (4.42)	<0.0001	6.92 (4.90)	0.0068	7.55 (5.47)	0.2021
Circulatory conditions (0–10)	1.06 (1.16)	1.23 (1.16)	<0.0001	1.02 (1.14)	0.1095	1.14 (1.26)	0.0644
Skeletomuscular conditions (0–6)	0.99 (0.99)	0.89 (0.95)	<0.0001	0.93 (0.99)	0.0160	1.04 (1.08)	0.8113
Mental health conditions (0–10)	1.34 (1.51)	1.22 (1.48)	<0.0001	1.41 (1.56)	0.1369	1.43 (1.70)	0.3961
Hearing and vision conditions (0–6)	0.61 (0.84)	0.48 (0.76)	<0.0001	0.58 (0.84)	0.2437	0.65 (0.88)	0.0559
Infectious diseases (0–4)	0.07 (0.27)	0.10 (0.32)	<0.0001	0.09 (0.31)	0.0028	0.11 (0.32)	0.0002
Kidney conditions (0–3)	0.04 (0.21)	0.04 (0.22)	0.0691	0.04 (0.20)	0.4836	0.05 (0.24)	0.1110
Gastrointestinal conditions (0–9)	0.86 (1.00)	0.74 (0.94)	<0.0001	0.79 (1.00)	0.0001	0.82 (1.02)	0.0059
Cancers (0–6)	0.23 (0.49)	0.11 (0.34)	<0.0001	0.14 (0.39)	<0.0001	0.15 (0.41)	<0.0001
Neurological conditions (0–12)	0.90 (1.19)	0.67 (0.99)	<0.0001	0.87 (1.17)	0.0359	0.98 (1.32)	0.1485
Other conditions (0–9)	1.07 (1.14)	1.05 (1.10)	0.5047	1.06 (1.16)	0.6481	1.18 (1.17)	0.0090
Pain intensity, past week (%)			<0.0001		<0.0001		<0.0001
No pain (0)	9.9	9.0		9.7		9.0	
Mild pain (1–3)	33.7	21.6		26.3		25.4	
Moderate pain (4–6)	30.6	30.6		30.1		29.6	
Severe pain (7–10)	25.7	38.8		33.9		36.0	
VA health care use, past year (%)			0.6526		<0.0001		0.0005
None	7.6	6.5		8.1		7.9	
Less than half of care	20.3	21.9		25.4		25.8	
More than half of care	26.6	25.2		23.7		24.5	
All care	45.5	46.5		42.8		41.9	
VA inpatient hospital stays, past year (%)			<0.0001		0.0002		0.0863
None	84.2	81.8		81.5		81.5	
1–3	13.4	15.3		15.2		16.3	
≥4	2.4	2.9		3.3		2.2	
Current prescription medications from VA (%)			<0.0001		0.8191		0.8113
None	15.8	10.7		15.3		15.8	
1–3	31.3	28.4		31.6		30.4	
4–6	25.0	29.2		25.8		25.0	
≥7	27.9	31.7		27.3		28.8	
Current prescription medications outside VA (%)			<0.0001		0.0064		0.0014
None	55.2	49.8		51.2		49.0	
1–3	30.1	32.3		33.1		31.5	
4–6	9.7	12.3		10.9		12.5	
≥7	5.0	5.6		4.8		7.0	

No. = number.

## Data Availability

Data will not be made available in order to comply with current VA privacy regulations.
